# Association between Remote Dielectric Sensing and Estimated Plasma Volume to Assess Body Fluid Distribution

**DOI:** 10.3390/jcm12020463

**Published:** 2023-01-06

**Authors:** Teruhiko Imamura, Toshihide Izumida, Nikhil Narang, Hiroshi Onoda, Masaki Nakagaito, Shuhei Tanaka, Makiko Nakamura, Ryuichi Ushijima, Hayato Fujioka, Kota Kakeshita, Koichiro Kinugawa

**Affiliations:** 1 Second Department of Internal Medicine, University of Toyama, Toyama 930-0194, Japan; 2 Advocate Christ Medical Center, Oak Lawn, IL 60453, USA

**Keywords:** congestion, heart failure, hemodynamics

## Abstract

Background: Pulmonary congestion is quantified by a remote dielectric sensing (ReDS^TM^) system, while systemic congestion is estimated by calculated plasma volume. The type of clinical patient profile as defined by the ReDS system and calculated plasma volume remains uncertain. Methods: Hospitalized patients with or without heart failure were included in this prospective study. On admission, ReDS values were measured and plasma volume status (PVS) was estimated using their body weight at the same time. Cutoffs of ReDS value and PVS were defined at 34% and −2.7%, respectively. The association between the two parameters was assessed. Results: A total of 482 patients (median 76 years, 288 men) were included. The median ReDS value was 28% (25%, 32%) and median PVS was −16.4% (−26.3%, −5.9%). Of the patients, 64 had high ReDS value (and low PVS) and 80 had high PVS (and low ReDS value). The high ReDS group had a higher prevalence of clinical heart failure with a more elevated echocardiographic E/e’ ratio, whereas the high PVS group had a higher prevalence of chronic kidney disease (*p* < 0.05 for all). Four out of a total of six patients with high ReDS value and high PVS had both heart failure and chronic kidney disease profiles. Conclusion: The combination of ReDS value and PVS was able to clinically stratify the types of body fluid distribution and patient profiles. Utilizing these tools may assist the clinician in constructing a therapeutic strategy for the at-risk hospitalized patient.

## 1. Introduction

In patients with chronic heart failure, residual pulmonary and systemic congestion following attempts at medical optimization is associated with an increased risk of morbidity and mortality [1]. Nevertheless, the practical and non-invasive tools that can accurately quantify the degree of pulmonary and systemic congestion in daily clinical practice are limited. Clinical examination often cannot accurately estimate pulmonary and systemic congestion compared to invasive right heart catheterization. It should be noted that routinely performing invasive hemodynamic assessments is not feasible for the majority of institutions that care for patients with heart failure [2]. 

The remote dielectric sensing (ReDS^TM^, Sensible Medical Innovations Ltd., Netanya, Israel) system is a non-invasive electro-magnetic technology that quantifies the degree of pulmonary congestion within one minute of application and without needing a high level of technical expertise [3]. ReDS values, whose normal range is suggested between 20% and 35%, have been shown to have a moderate correlation (i.e., approximate r value of 0.6) with other modalities, including chest-computed tomography and pulmonary capillary wedge pressure [4,5,6]. 

Recently, a novel technique to non-invasively estimate plasma volume (PV) using hematocrit (Ht) and body weight (BW), has been made commercially available [7]. PV is considered to represent the amount of extra-vascular fluid. This modality was validated using scintigraphy tests and the ability to accurately estimate systemic congestion has been shown in recently published clinical studies [8,9]. 

The association between ReDS and PV quantification has not been rigorously analyzed thus far. The combination of these two methods may prove effective in assessing total body volume distribution, allowing the clinician to construct therapeutic strategies for each patient. In this study, we evaluated the volume distribution profile of hospitalized patients with cardiovascular diseases using both the ReDS system and estimated PV. 

## 2. Methods

### 2.1. Participant Selection

Patients who were hospitalized for treatment of their cardiovascular diseases including heart failure were considered to be included in this cross-sectional study. Patients were assumed to have heart failure when they had a history of heart failure or ongoing heart failure, which was diagnosed by Framingham’s criteria, irrespective of their left ventricular ejection fraction. Chronic kidney disease was defined as estimated glomerular filtration ratio <60 mL/min/1.73 m^2^ or urinary albumin excretion ≥300 mg/day. 

On admission, ReDS measurements were performed as detailed below. PV was calculated as detailed below using Ht and BW data on admission. 

Patients who were unable to wear an ReDS system due to body size (for example, body mass index > 35), or those with lung pathology including pneumonia and lung cancer, were not eligible to undergo ReDS measurements. Patients who had BW data that was not collected on admission were also excluded. Informed consent was obtained from all participants beforehand. The institutional ethical review board approved the study protocol. 

### 2.2. ReDS Measurement

ReDS measurements were performed on all participants. ReDS employs low-power electromagnetic signals emitted between two sensors embedded on a wearable device and can quantify lung fluid amount within one minute non-invasively with normal breathing mechanics [3]. The manufacturer-proposed normal range for the ReDS value is between 25% and 34%. 

### 2.3. PV Estimation

All participants received BW, body height, and Ht measurements on admission. The PV was estimated by calculating the Hakim formula: PV (L) = [1 − Ht (%)] × [a × (b × (lean body mass))], in which a = 1530 for males and 864 for females, and b = 41.0 for males and 47.2 for females. The lean body mass was calculated as follows: 0.33 × BW (kg) + 0.34 × [body height (cm)] − 29.5 for males and 0.30 × BW (kg) + 0.42 × [body height (cm)] − 43.3 for females [7]. 

PV status (PVS) was calculated as follows: PVS (%) = [((estimated PV) − (ideal PV))/(ideal PV)] × 100. Ideal PV (L) = a × BW (kg), in which a = 39 for males and 40 for females. 

### 2.4. Other Variables

Laboratory data including plasma B-type natriuretic peptide and transthoracic echocardiography data were obtained on admission according to the standard manner. 

### 2.5. Statistical Methods

Continuous variables were calculated as a median and interquartile range. Categorical variables were calculated as numbers and percentages. The correlation between ReDS values and estimated PVS was assessed using Pearson’s correlation. 

A cutoff of ReDS value was defined at 34%. Patients with ReDS values above this cutoff were classified as having clinically significant pulmonary congestion. A cutoff of PVS was defined at −2.7% [10]. Although a definite cutoff of PVS has not yet been established, the mean value of PVS in the healthy cohort was −2.7%. Patients with a PVS above this cutoff were assumed to have significant systemic congestion. Patients were stratified into four groups using these two cutoffs for both ReDS and PVS. 

All analyses were performed in SPSS Statistics 23.0 software (IBM Corp, Armonk, NY, USA), and two-tailed p values less than 0.05 were assumed to be significant.

## 3. Results

### 3.1. Baseline Characteristics

A total of 500 hospitalized patients were considered for study inclusion. Of these, 18 patients with pneumonia were excluded. Eventually, 482 patients were included in the final study cohort. Median age was 76 (69, 82) years old and 288 (60%) were men (Table 1). Of these, 136 (28%) had a diagnosis of heart failure (including 33 preserved ejection fraction) and 319 (66%) had chronic kidney disease. 

### 3.2. Association between ReDS Value and PVS

**Figure 1:**
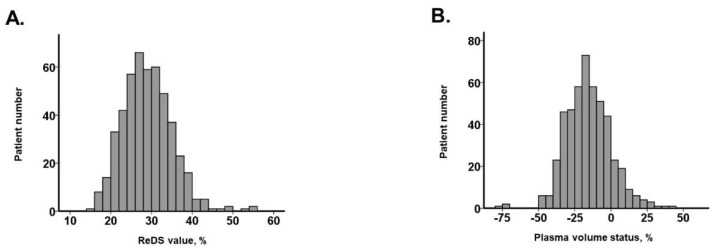
Distribution of ReDS value (**A**) and PVS (**B**).

**Figure 2:**
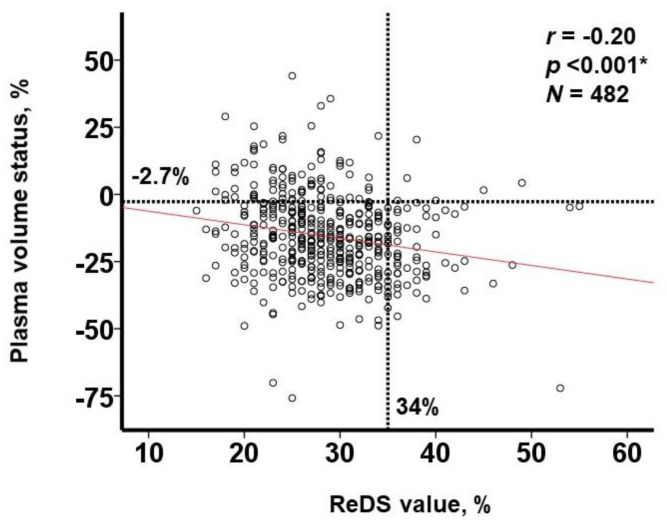
Correlation between ReDS value and PVS. Cutoffs for ReDS value and PVS were defined at 34% and −2.7%, respectively. * *p* < 0.05 by Pearson’s correlation.

Median ReDS value was 28% (25%, 32%) and median PVS was −16.4% (−26.3%, −5.9%). These values were distributed widely (Figure 1A,B). There was a significant but weak correlation between the two values (r = −0.20, *p* < 0.001; N = 482; Figure 2). Of these, 70 patients had ReDS values >34% and 86 patients had PVS >−2.7%. Most of the patients (69%) had a low ReDS value and low PVS. Only six patients had a high ReDS value and high PVS. 

### 3.3. Profile of Those with High ReDS Value and/or High PVS

Out of all the patients, 150 (31%) had a high ReDS value and/or high PVS. They tended to have heart failure (33% versus 26%) and chronic kidney disease (71% versus 64%) more frequently than those with a low ReDS value and low PVS (*p* = 0.059 and *p* = 0.097, respectively; Table 1). They also had a higher plasma level of B-type natriuretic peptide and a higher echocardiographic E/e’ ratio than those with a low ReDS value and low PVS (*p* < 0.001 and *p* = 0.013, respectively). They also had higher prevalence of diabetes mellitus, anemia, and hypoalbuminemia than those with a low ReDS value and low PVS (*p* < 0.05 for all). 

### 3.4. Profile Comparison between High ReDS Group and High PVS Group

Out of all the patients, 64 had a high ReDS value (and low PVS) and 80 had a high PVS (and low ReDS value) (Figure 2). The high ReDS group was younger and had a more elevated echocardiographic E/e’ ratio than the high PVS group (*p* < 0.05 for both; Table 2). The high PVS group was older and had a higher prevalence of chronic kidney disease than the high ReDS group (*p* < 0.05 for all; Table 2). They also had a higher prevalence of anemia, hypoalbuminemia, and a higher plasma B-type natriuretic peptide level than the high ReDS group (*p* < 0.05 for all).

### 3.5. Profile of Patients with High ReDS and High PVS

Only six patients had a high ReDS and high PVS (median 78 [73, 87] years old, five men). Of these, four patients (67%) had both heart failure and chronic kidney disease and one patient had chronic kidney disease alone. Median ReDS value was 39% (37%, 45%) and median PVS was 2.9% (−2.2%, 6.1%). Median echocardiographic E/e’ ratio was 13.2 (10.7, 14.7) and median serum creatinine was 3.0 (0.9, 5.0) mg/dL. 

### 3.6. Profile of Patients with and without Heart Failure

Several key parameters were compared between those with and without heart failure (Table 3). Plasma B-type natriuretic peptide levels and echocardiographic E/e’ ratio were higher in the heart failure group, whereas ReDS values and PVS were not statistically different between the two groups.

Among 136 heart failure patients, ReDS values did not have any correlations with ejection fraction (*p* = 0.15) and B-type natriuretic peptide levels (*p* = 0.11). The PVS did not have correlation with ejection fraction (*p* = 0.30), but had a significant but weak correlation with B-type natriuretic peptide (*p* = 0.032, r = 0.18).

## 4. Discussion

We investigated patients’ profiles stratified by ReDS values and PVS among those who were hospitalized for treatment for cardiovascular diseases. The high ReDS (and low PVS) group had a more elevated echocardiographic E/e’ ratio, whereas the high PVS (and low ReDS) group had a higher prevalence of chronic kidney disease. Four out of six patients satisfying both a high ReDS value and high PVS had both heart failure and chronic kidney disease.

### 4.1. Rationale for Utilizing ReDS System and PVS

Assessment of pulmonary congestion can be challenging. The ReDS system can quantify the degree of pulmonary congestion with reasonable accuracy when compared to other modalities, including high-resolution computed tomography [5], right heart catheterization [4], and lung ultrasound [11].

The methodology to estimate the PVS by using the Hakim formula, as a surrogate of systemic congestion, was demonstrated and validated by previous studies [8,9]. Its prognostic impact was demonstrated in cohorts of patients with severe aortic stenosis [12], ischemic heart disease requiring coronary artery bypass surgery [13], and advanced heart failure requiring a durable left ventricular assist device [14]. Given the clinical conditions where quantifying systemic congestion was observed to be feasible, we sought to estimate a comprehensive congestion profile in a cohort with a low likelihood of clinical congestion in addition to those who have a higher likelihood of a clinically abnormal congestion profile.

### 4.2. Patients’ Profiles Stratified by ReDS Value and PVS

No positive correlation existed between ReDS values and PVS. ReDS values overall did not have a strong correlation with PVS. Thus, ReDS cannot be an alternative to PVS, and vice versa. In other words, the combination of both modalities may be more practical to accurately understand body fluid distribution than each alone.

Patients’ clinical profiles were uniquely characterized by combination of both modalities. Patients with a high ReDS value had a higher prevalence of heart failure etiology and a more elevated echocardiographic E/e’ ratio. The elevations of intra-cardiac filling pressures due to impaired cardiac function often coincide with pulmonary congestion, though may not manifest as systemic congestion [15]. Prior analyses have shown that ReDS value moderately correlates with pulmonary capillary artery pressure, but does not strongly correlate with central venous pressure [4].

Patients with a high PVS tended to have chronic kidney disease and a lower echocardiographic E/e’ ratio. These patients may therefore have systemic volume overload due to impaired renal function [16], and may not have pulmonary congestion. They had higher plasma B-type natriuretic peptide levels than those with high ReDS, probably due to a higher prevalence of chronic kidney disease in this cohort.

### 4.3. Clinical Implication

A combination of both modalities may be practical to estimate patients’ profiles and determine therapeutic strategy, instead of either modality alone, particularly at the time of admission when comprehensive clinical assessments ha not yet been completed.

When ReDS values are high (and PVS is low), these patients have a likelihood of clinical heart failure with increased intra-cardiac filling pressures and will benefit from vasodilators first, followed by diuretics therapy. When PVS values are high (and the ReDS value is low), these patients may have volume overload due to chronic kidney disease. Diuretics may be beneficial instead of heart failure-specific therapies to address systemic congestion. When patients had high ReDS values and high PVS, they would have both pulmonary and systemic congestion probably due to heart failure and renal impairment accompanying hemodynamic deterioration. Aggressive dehydration therapy using multiple therapeutic modalities would be required.

On the contrary, ReDS values and PVS were not significantly stratified by the existence of heart failure. These are not diagnostic tools. Instead, we can quantify the amount and distribution of body fluid and adjust medications by referencing their combination, irrespective of any etiologies.

### 4.4. Study Limitations

This study is not without limitations. It consists of a moderate sample-sized cohort collected from a single center. Our cohort included relatively more patients with chronic kidney disease compared with other studies probably due to a relatively higher age and multiple comorbidities. Our findings may not simply be adopted to other cohorts given the lack of validation thus far. Further larger-scale multi-institutional studies are warranted to validate and generalize our findings.

Given the lack of any cutoffs of PVS, we used a mean value of the healthy cohort. A high PVS above the cutoff in this study may not indicate clinically significant systemic congestion. Thus, PVS may not be a good diagnostic tool thus far. The cutoff of PVS with any clinical implications needs investigation in future studies. We proposed a therapeutic strategy by referencing the combination of a ReDS value and PVS, though prospective validation of these modalities in guiding therapy needs further investigation.

### 4.5. Conclusions

The combination of ReDS values and PVS was able to better classify clinical profiles often associated with either pulmonary congestion, systemic congestion, or neither. These modalities may be useful adjunctive diagnostic tools to consider when developing a therapeutic strategy.

## Figures and Tables

**Table 1:** Baseline characteristics.

	Total (N = 482)	High ReDS and/or High PVS (N = 150)	Low ReDS and Low PVS (N = 332)	*p* Value
Demographics				
Age, years	76 (69, 82)	77 (71, 84)	76 (68, 81)	0.021 *
Men	288 (60%)	106 (71%)	182 (55%)	0.001 *
Body mass index	22.8 (20.5, 25.2)	21.3 (18.8, 23.8)	23.3 (21.4, 25.8)	<0.001 *
Systolic blood pressure, mmHg	123 (106, 137)	120 (102, 136)	125 (108, 138)	0.15
Heart rate, bpm	73 (65, 84)	74 (66, 83)	72 (65, 84)	0.89
Comorbidity				
Heart failure	136 (28%)	50 (33%)	86 (26%)	0.059
Hypertension	356 (74%)	115 (77%)	241 (73%)	0.20
Dyslipidemia	264 (55%)	80 (53%)	184 (55%)	0.37
Diabetes mellitus	170 (35%)	66 (44%)	104 (31%)	0.005 *
Chronic obstructive pulmonary disease	21 (4%)	7 (5%)	14 (4%)	0.32
Persistent atrial fibrillation	77 (16%)	21 (14%)	55 (17%)	0.47
Paroxysmal atrial fibrillation	94 (20%)	31 (21%)	61 (18%)	0.55
Chronic kidney disease	319 (66%)	106 (71%)	213 (64%)	0.097
History of stroke	90 (19%)	32 (21%)	58 (17%)	0.19
History of coronary intervention	115 (24%)	43 (29%)	72 (22%)	0.062
Valvular disease	160 (33%)	68 (45%)	92 (28%)	<0.001 *
Laboratory data				
Hemoglobin, g/dL	12.6 (11.2, 13.9)	11.9 (10.2, 13.0)	13.0 (11.5, 14.2)	<0.001 *
Serum albumin, g/dL	3.9 (3.6, 4.2)	3.7 (3.4, 4.0)	3.9 (3.7, 4.2)	<0.001 *
Serum GOT, IU/L	24 (12, 33)	27 (13, 35)	23 (12, 34)	0.076
Serum GPT, IU/L	23 (11, 34)	25 (12, 34)	22 (11, 33)	0.068
Serum creatinine, mg/mL	1.0 (0.8, 1.5)	1.1 (0.8, 2.0)	1.0 (0.9, 1.3)	0.002 *
Serum sodium, mg/dL	140 (138, 141)	139 (137, 141)	140 (138, 141)	0.28
Plasma B-type natriuretic peptide, pg/mL	105 (35, 283)	174 (71, 498)	84 (27, 196)	<0.001 *
Echocardiography				
Left ventricular end-diastolic diameter, mm	48 (44, 55)	49 (44, 57)	48 (44, 54)	0.15
Left ventricular ejection fraction, %	63 (51, 71)	62 (50, 71)	63 (52, 72)	0.70
Left atrial diameter, mm	41 (35, 50)	43 (35, 52)	41 (35, 49)	0.072
E/e’ ratio	8.5 (6.2, 9.8)	11.2 (9.5, 12.9)	7.6 (5.6, 9.2)	0.013 *
ReDS value, %	28 (25, 32)	32 (25, 38)	28 (25, 31)	<0.001 *
Plasma volume status, %	−16.4 (−26.3, −5.9)	−1.5 (−19.5, 6.1)	−18.7 (−27.6, −11.7)	<0.001 *

Continuous variables are stated as median (25% interquartile, 75% interquartile) and compared between the two groups using the Mann−Whitney U test. Categorical variables are stated as numbers and percentage and compared between the two groups using Fischer’s exact test. * *p* < 0.05. GOT, glutamate oxaloacetate transaminase; GPT, glutamate pyruvate transaminase; ReDS, remote dielectric sensing; PVS, plasma volume status.

**Table 2:** Comparison between high ReDS group and high PVS group.

	High ReDS (N = 64)	High PVS (N = 80)	*p* Value
Demographics			
Age, years	73 (63, 82)	79 (73, 84)	0.002 *
Men	35 (55%)	66 (83%)	<0.001 *
Body mass index	24.3 (21.9, 28.6)	19.4 (17.6, 20.8)	<0.001 *
Systolic blood pressure, mmHg	121 (104, 137)	119 (102, 139)	0.78
Heart rate, bpm	77 (64, 90)	70 (65, 82)	0.16
Comorbidity			
Heart failure	21 (33%)	25 (31%)	0.49
Hypertension	44 (69%)	66 (83%)	0.042 *
Dyslipidemia	33 (52%)	44 (55%)	0.40
Diabetes mellitus	24 (38%)	38 (48%)	0.15
Chronic obstructive pulmonary disease	3 (5%)	4 (5%)	0.67
Persistent atrial fibrillation	11 (17%)	10 (13%)	0.67
Paroxysmal atrial fibrillation	17 (27%)	14 (18%)	0.37
Chronic kidney disease	38 (59%)	63 (79%)	0.010 *
History of stroke	12 (19%)	18 (23%)	0.37
History of coronary intervention	16 (25%)	25 (31%)	0.26
Valvular disease	26 (41%)	39 (49%)	0.21
Laboratory data			
Hemoglobin, g/dL	12.6 (11.2, 13.6)	11.2 (10.1, 12.4)	<0.001 *
Serum albumin, g/dL	3.9 (3.5, 4.2)	3.7 (3.3, 3.9)	0.012 *
Serum GOT, IU/L	26 (14, 34)	29 (14, 41)	0.076
Serum GPT, IU/L	24 (13, 33)	27 (13, 38)	0.066
Serum creatinine, mg/mL	0.9 (0.7, 1.4)	1.5 (0.9, 3.9)	<0.001 *
Serum sodium, mg/dL	140 (138, 142)	139 (137, 141)	0.18
Plasma B-type natriuretic peptide, pg/mL	124 (63, 422)	216 (93, 501)	0.034 *
Echocardiography			
Left ventricular end-diastolic diameter, mm	49 (45, 58)	48 (43, 57)	0.59
Left ventricular ejection fraction, %	62 (52, 71)	65 (50, 72)	0.59
Left atrial diameter, mm	44 (37, 54)	41 (34, 51)	0.24
E/e’ ratio	12.1 (10.2, 13.6)	9.8 (7.7, 10.6)	0.025 *
ReDS value, %	37 (36, 39)	25 (21, 28)	<0.001 *
Plasma volume status, %	−24.0 (−31.3, −12.2)	5.1 (0.6, 11.4)	<0.001 *

Continuous variables are stated as median (25% interquartile, 75% interquartile) and compared between the two groups using the Mann−Whitney U test. Categorical variables are stated as numbers and percentage and compared between the two groups using Fischer’s exact test. * *p* < 0.05. GOT, glutamate oxaloacetate transaminase; GPT, glutamate pyruvate transaminase; ReDS, remote dielectric sensing; PVS, plasma volume status.

**Table 3:** Comparison between those with and without heart failure.

	Heart Failure (N = 136)	No Heart Failure (N = 346)	*p* Value
Serum creatinine, mg/mL	1.1 (0.8, 1.5)	1.0 (0.8, 1.4)	<0.001 *
Plasma B-type natriuretic peptide, pg/mL	293 (116, 591)	80 (26, 208)	<0.001 *
E/e’ ratio	10.4 (8.4, 12.2)	9.1 (7.7, 10.9)	0.008 *
ReDS value, %	28 (25, 34)	29 (25, 33)	0.30
Plasma volume status, %	−17.1 (−25.6, −4.4)	−15.8 (−25.7, −7.4)	0.18

Continuous variables are stated as median (25% interquartile, 75% interquartile) and compared between the two groups using Mann−Whitney U test. * *p* < 0.05. ReDS, remote dielectric sensing; PVS, plasma volume status.

## Data Availability

Data are available upon reasonable requests.
